# Expression profiles and prognostic significance of WNT family members in glioma via bioinformatic analysis

**DOI:** 10.1042/BSR20194255

**Published:** 2020-03-27

**Authors:** Anqi Xu, Huiping Yang, Kunjie Gao, Zhengming Zhan, Zibin Song, Tengyue Huang, Ye Song

**Affiliations:** 1 Department of Neurosurgery, Nanfang Hospital, Southern Medical University, Guangzhou 510515, Guangdong, P.R. China; 2 The First Clinical Medical Institute of Southern Medical University, Guangzhou 510515, P.R. China; 3 Gannan Medical University, Ganzhou 341000, Jiang xi, P.R. China

**Keywords:** bioinformatics, expression, glioma, prognosis, WNT

## Abstract

**Aims:** The dysregulation and essential role of WNTs in glioma have been widely implicated. However, there is a paucity of literature on the expression status of all the 19 WNTs in glioma. Our study was aimed to evaluate the expression and prognostic values of the 19 WNTs in glioma. **Methods:** mRNA expression and clinical data were retrieved from the Cancer Genome Atlas (TCGA) database, Chinese Glioma Genome Atlas (CGGA), GTEx and ONCOMINE databases. The 50 frequent neighbor genes of WNT5A and WNT10B were shown with PPI network, Gene Ontology (GO) and Kyoto Encyclopedia of Genes and Genomes (KEGG) analyses. **Results:** We found that the mRNA expression of WNT5A was significantly higher in glioma; however, the WNT10B expression was significantly lower in glioma. Furthermore, the expression of WNT5A and WNT10B was associated with the clinicopathology of glioma. The survival analysis revealed that the higher expressions of WNT5A and WNT16 were associated poor overall survival (OS) in patients with glioma. Conversely, overexpression of WNT3, WNT5B, and WNT10B was associated with better OS. Finally, Go and KEGG analysis revealed WNT5A was associated with multiple signal translations, and crucial oncogenes (EGFR and MDM2) and 2 important tumor suppressors (PTEN and IKN4a/ARF) were found closely correlated with WNT5A in glioma. **Conclusion:** Among 19WNTs, WNT5A can serve as a candidate to diagnose and therapy glioma, while WNT10B might be valuable for anti-glioma research. The presumed direction was provided to explore the relation of WNTs signal and multiple pathways in glioma.

## Introduction

Glioma represents the most frequent primary intracranial neoplasms and is characterized by aggressive growth and poor prognosis [1]. The WHO classification divides glioma originating from a glial cell into 4 (I–IV) grades based on the malignant degree [1]. Glioblastoma (GBM; WHO grade IV), which constitutes 54.9% of all gliomas, is the most aggressive and highly invasive type of glioma. For the patients with GBM, the median overall survival is merely 12–15 months and the 5-year survival rate remains less than 5% [2,3]. As the heterogeneity represents a major challenge in precise diagnosis and therapy of glioma [4], the molecular signatures of glioma are urgent to be explored for diagnosis and therapy [5].

In the hundreds of previous studies, 8 members of 19 WNTs, WNT1, 2, 2B, 3A, 5A, 6, 7A and 7B, were showed being related with glioma development [4,6–9]. Nowadays, increasing evidences report that WNTs play essential role in glioma progress [2]. WNT1 has been suggested to be overexpressed with a paradoxical role in glioma [10,11]. WNT2 knockdown in glioma cells significantly suppressed growth and associated with the decrease of PI3K/p-AKT expression *in vitro* and *in vivo* [12]. Furthermore, WNT1 and WNT3A were found to increase glioma-derived stem-like cells chemo-resistance, proliferation and migration [11]. In addition, WNT5A was found to induce rapid growth and migration of glioma and associated with the presence of tumor-associated microglia [8,9,13,14]. WNT6 expression was shown to increase the features of glioma aggressiveness [6]. WNT7A and WNT7B was shown to regulate glioma-vascular interactions [7]. Collectively, these pieces of evidence indicate that targeting WNT signaling might be an effective therapeutic strategy against glioma.

Nineteen WNTs of *Homo sapiens* are numbered in the order of discovery: WNT1, 2, 2B, 3, 3A, 4, 5A, 5B, 6, 7A, 7B, 8A, 8B, 9A, 9B, 10A, 10B, 11 and 16 [15,16]. WNT signaling cascade was an evolutionarily conserved pathway [17]. Abnormality of WNT signaling cascade has been found in many cancers. WNTs are known to function via three WNT signaling pathways, including the canonical WNT/β-catenin pathway, and the two non-canonical pathways such as WNT/PCP and WNT/Ca^2+^ pathways. Accumulating evidences indicate that 11 members of WNT1, 2, 2B, 3, 3A, 7A, 7B, 9A, 9B, 10A and 10B are predominant ligands of canonical pathway [11,18–23]; WNT7A and WNT7B are also the ligands of WNT/PCP pathway [24–26]. WNT5A and 5B are mainly considered as the ligands of WNT/Ca^2+^pathway and take part in the canonical pathway as well [20,27]. Besides, six WNT members, WNT4, 6, 8A, 8B, 11 and 16, remain largely unknown for which specific pathway they function [21,28,29]. However, which WNT of 19 WNT family members are the most potential to be biomarkers of glioma and the way they take part in glioma progress are still not clear.

In the present study, we extensively used bioinformatic approaches to analyze the expression profiles and evaluate the prognostic significance of all the 19 members of the WNT family in glioma with publicly available data from TCGA, CGGA, CCLE and other databases. The PPI network, GO and KEGG analyses were performed to analyze the biofunction and molecular mechanism of several essential WNTs in glioma. Therefore, we could find out the most reliable prognostic markers and therapeutic strategies against glioma in 19 WNTs.

## Materials and methods

### ONCOMINE database

ONCOMINE (www.oncomine.org) is currently the world’s largest online data-mining platform including data of over 20 cancers from TCGA and GEO databases [30]. The transcriptional mRNA expression data of 19 WNTs across different cancers along with corresponding normal tissues was retrieved from ONCOMINE database. The *P*-value was set up at 0.01.

### Broad Institute Cancer Cell Line Encyclopedia (CCLE) database

The CCLE (https://www.broadinstitute.org/ccle) database was used to retrieve the mRNA expression of WNTs in various cancer cell lines, and the data are verified and illustrated as box plots [31]. In addition, the mRNA expression of 19 WNTs was compared in multiple well-known glioma cell lines.

### The Cancer Genome Atlas (TCGA) database and Chinese Glioma Genome Atlas (CGGA) database

The TCGA database is a comprehensive and coordinated project, including information about sequencing and pathological data of more than 30 types of human tumors. Clinicopathological parameters and WNTs’ mRNA expression from 665 patients with glioma (GBM:156, LGG:509) were downloaded for our study. The CGGA database is a free used platform to investigate glioma from Chinese cohorts including over 600 glioma samples of patients. The mRNA microarray of 301 patients and matched clinical data were retrieved in the present study. The clinicopathological correlation and prognostic value of WNTs’ mRNA expression in glioma were accepted only when the results are statistically significant in both databases.

### Gene Expression Profiling Interactive Analysis (GEPIA)

In our study, the mRNA expression data of 163 GBM samples and 518 LGG samples were retrieved from TCGA database, and the mRNA expression data of 207 NB samples was retrieved form GTEx database. For analysis, we utilized GEPIA to compare mRNA expression of WNTs between glioma and NB tissues. Furthermore, we performed the survival analysis of 19 WNTs in patients with glioma [32].

### c-BioPortal

c-BioPortal (www.cbioportal.org) is an online open-access website to easily explore, visualize and analyze the multidimensional genomic data of cancers. Following the c-BioPortal’s online instruction, 50 frequent neighbor genes of WNT5A and WNT10B were determined as summarized in Table 2.

### PPI network and module analysis

STRING (https://string-db.org/) was used to make PPI network of WNT5A/10B and 50 frequent neighbor genes. Then, Cytoscape was applied to examine the potential correlation between these genes. In addition, the MCODE app and BONGO app in Cytoscape were used to check modules of the PPI network (degree cutoff = 2, max. Depth = 100, k-core = 2, and node score cutoff = 0.2).

### Gene Ontology (GO) and Kyoto Encyclopedia of Genes and Genomes (KEGG) analysis

GO analysis predicted the functions of WNT5A and 10B and in glioma on the basis of three aspects, including molecular function, biological processes and biological pathway. KEGG analysis showed the important role of WNT5A, visualized with the online website DAVID (https://david.ncifcrf.gov/summary.jsp).

### Statistical methods

IMB SPSS 22.0 and GraphPad Prism 7.0 software were used to analyze statistical data and plot the graphs. All values were expressed as means ± standard deviations (SD). *P* < 0.05 was considered to be statistically significant. A Student’s *t-*test was used to assess the differences between two groups. Different grade of malignant glioma was analyzed by one-way analysis of variance (ANOVA) followed by Dunnett test. The Kaplan–Meier method was used to plot survival curves and compared using the log-rank test.

## Results

### mRNA expression of WNTs in glioma compared with NB tissue

**Figure 1:**
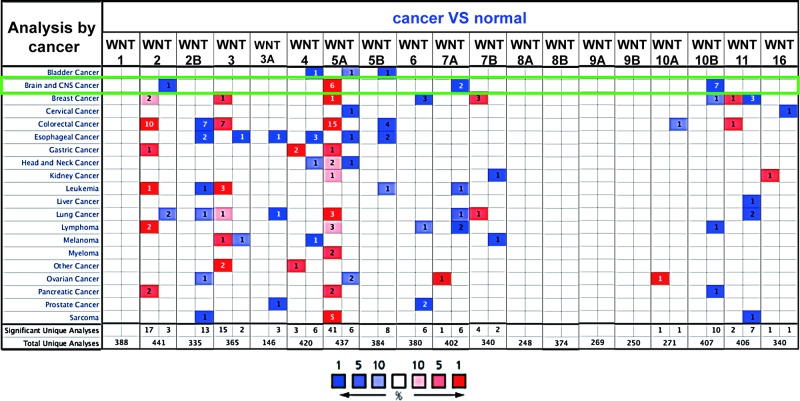
Transcriptional expression of 19 WNTs in different cancer types using the ONCOMINE database. mRNA overexpression (red) or down-regulation (blue)

Thus far, 19 WNT ligands have been identified in *Homo sapiens*. By analyzing the ONCOMINE database, the expression profiles of WNTs were analyzed across 20 cancers and compared with corresponding normal tissues. The higher level of WNT5A mRNA expression is elevated in most cancers. For CNS tumor, higher expression of WNT5A, lower expression of WNT7A and lower expression of WNT10B were found in glioma compared with NB tissues (Figure 1).

**Figure 2:**
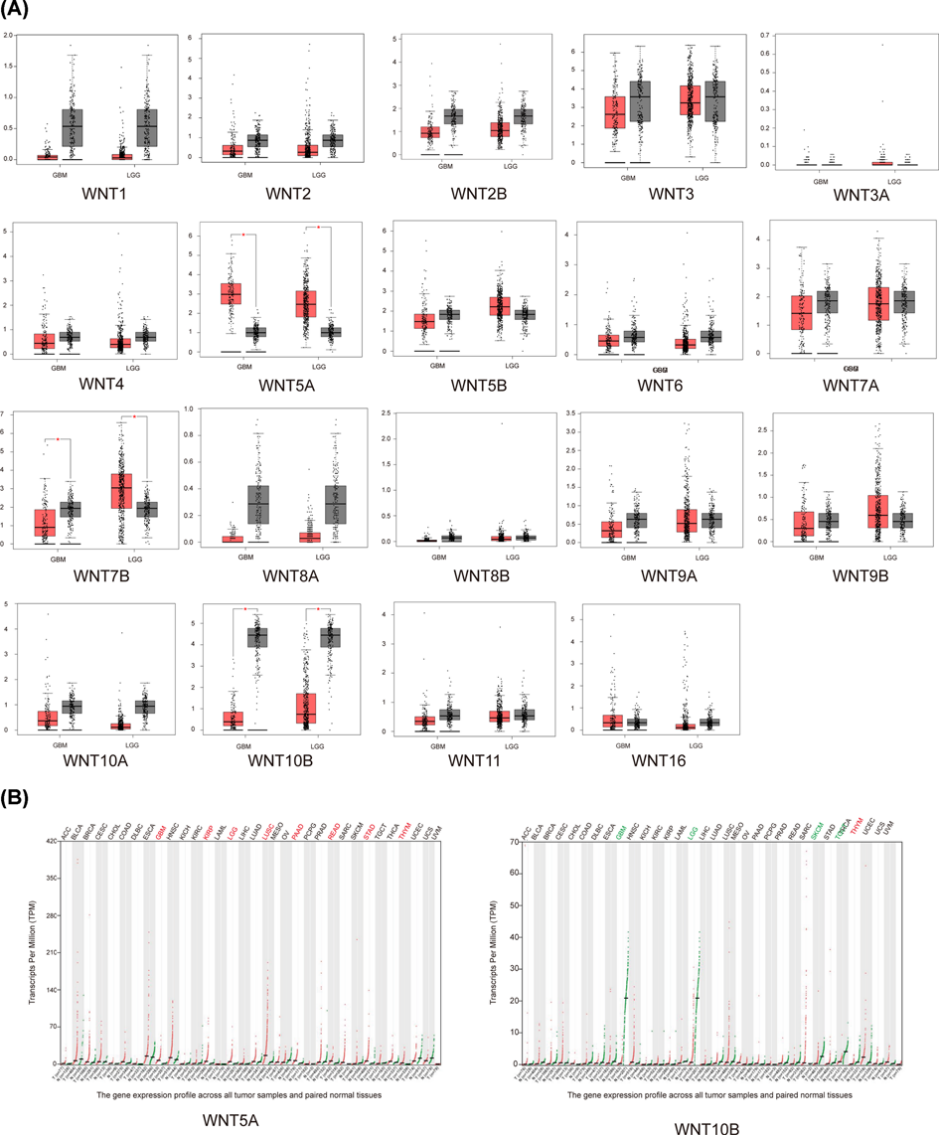
mRNA expression of 19 WNT genes in glioma including GBM and LGG compared with NB tissue using GEPIA (**A**) Statistically significant higher expression of WNT5A and lower expression of WNT10B in both GBM and LGG compared with NB (GBM: *n*= 163, LGG: 518, NB: *n*= 207) analyzed with TCGA and GTEx datasets (*P* <0.05). (**B**) Higher expression of WNT5A and lower expression of WNT10B in GBM and LGG compared with other human tumors.

Glioblastoma (GBM; WHO grade IV) was the most malignant of all glioma, the others were low grade glioma (LGG; WHO grade I–III). We analyzed 19 WNTs expression in GBM and LGG compared with NB tissues using GEPIA website. The result suggested that only higher expression of WNT5A and lower expression of WNT10B were detected with statistical significance (Figure 2A). In multiple human tumors, significantly higher mRNA expression of WNT5A and lower mRNA expression of WNT10B in GBM and LGG were also shown (Figure 2B).

### mRNA expression of WNTs in glioma cell lines

**Figure 3:**
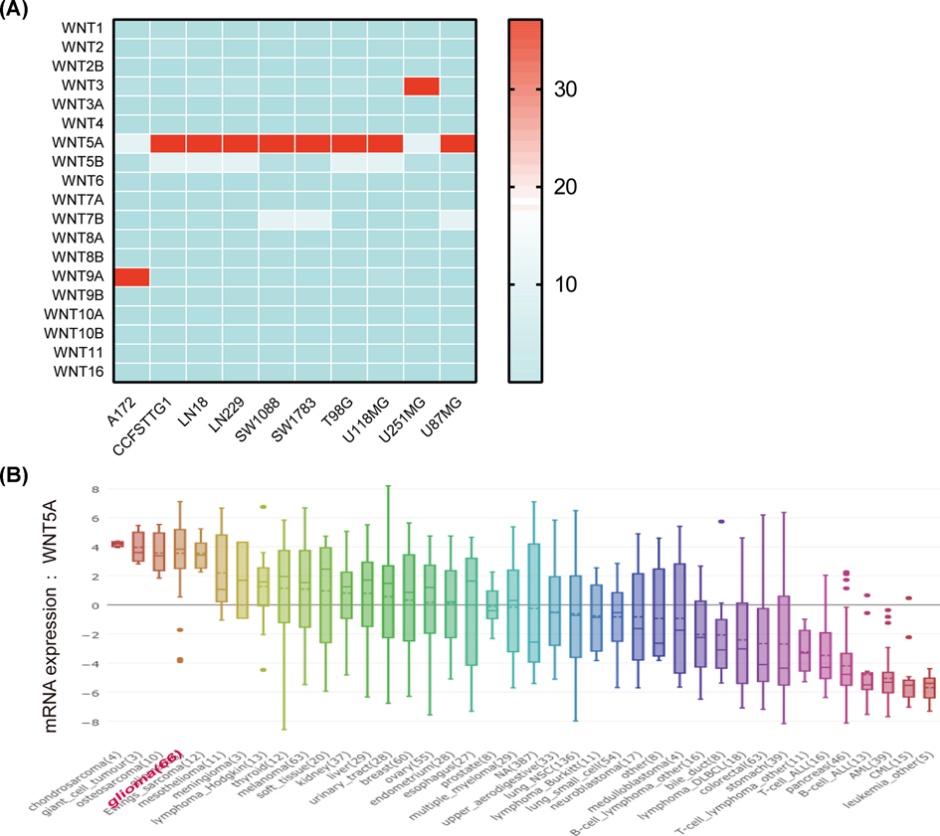
The expression of WNTs in glioma cell lines using CCLE database (**A**) Among 19 WNTs, higher expression of WNT5A in multiple widely used glioma cell lines. (**B**) Significantly higher expression of WNT5A in glioma compared with other cancer cell lines.

Then, we analyzed the mRNA expression of WNTs in glioma cell lines by assembling the CCLE database. We expanded the procedure of detailed annotation in preclinical human glioma models. WNT5A was differentially higher expressed in glioma cell lines among 19 WNTs, implying that WNT5A might play distinct functions in glioma (Figure 3A). CCLE analysis was also revealed that WNT5A mRNA expression was significantly up-regulated in glioma compared with other human cancers (Figure 3B).

### The prognostic values of WNTs in glioma

**Figure 4:**
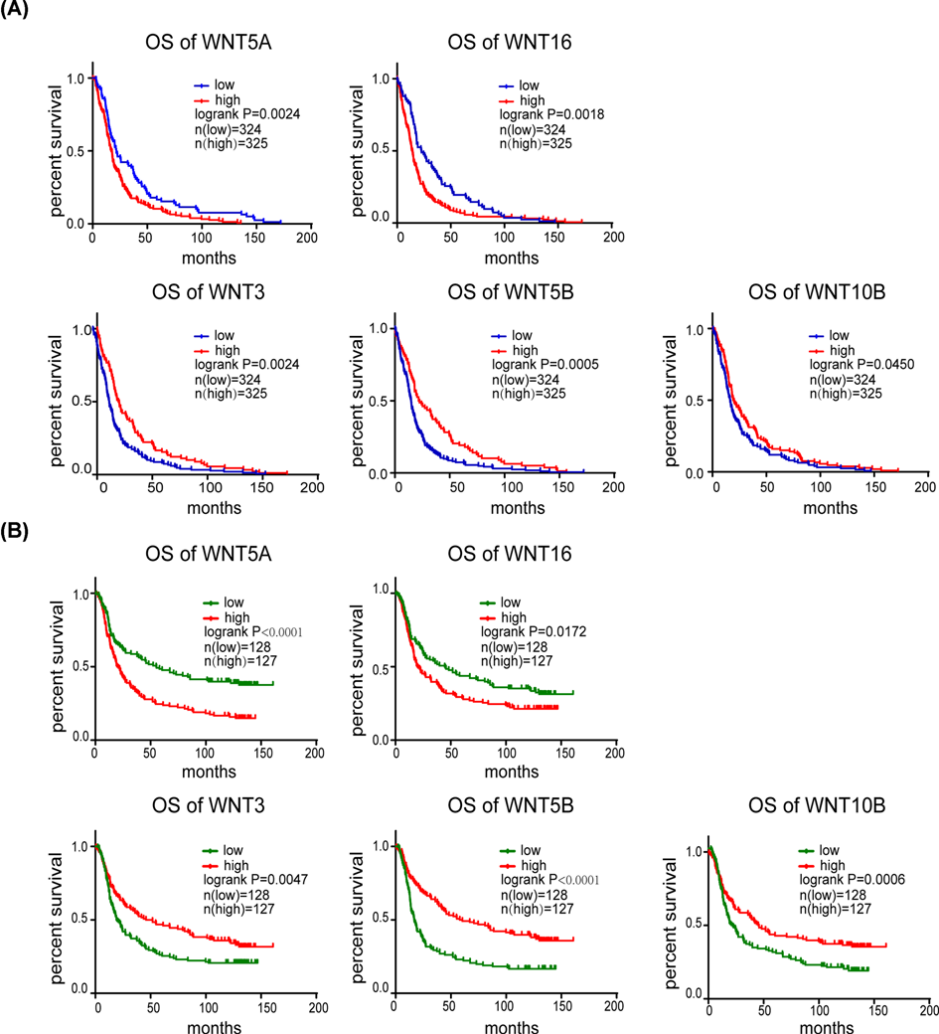
The prognostic value of the mRNA level of WNTs in patients with glioma analyzed with TCGA and CGGA databases (**A**) The prognostic value of the mRNA level of WNTS in patients with glioma analyzed using the TCGA database. (**B**) The prognostic value of the mRNA level of WNTS in patients with glioma analyzed using the CGGA database.

To investigate the prognostics significance of WNTs in glioma, we further analyzed mRNA expression data of WNTs with clinical data using Kaplan–Meier survival analysis. Higher mRNA levels of WNT5A and WNT16 were significantly associated with poor OS in patients with glioma (Figure 4A,B). Conversely, the increased mRNA expression levels of WNT3, WNT5B and WNT10B were correlated with better OS in patients with glioma (Figure 4A,B).

### Clinicopathological correlation of WNTs mRNA expression in patients with glioma

**Figure 5:**
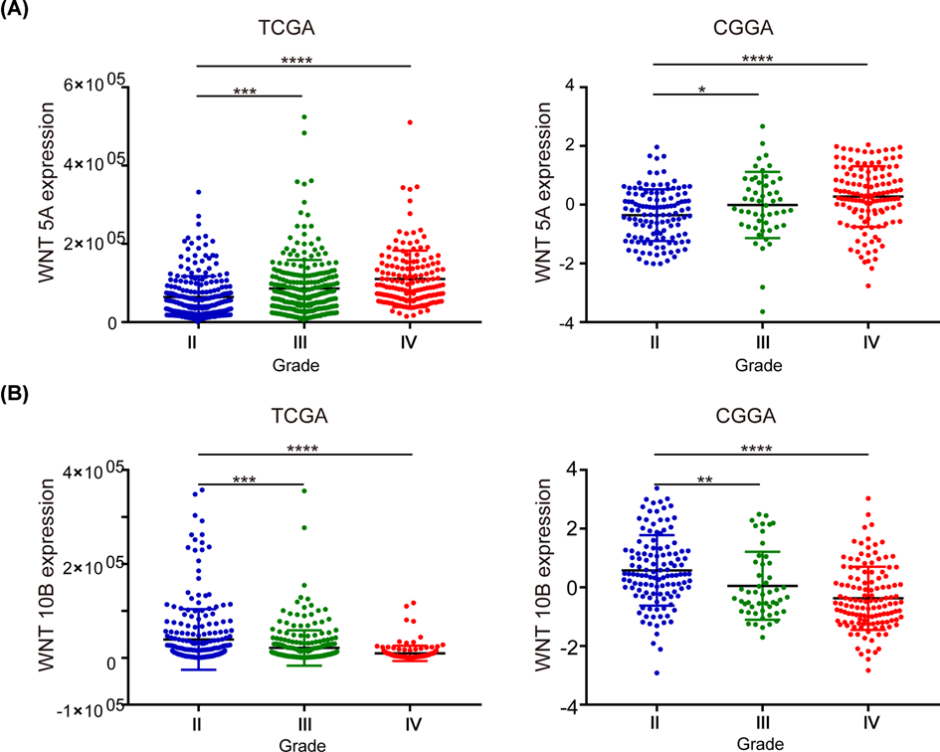
Correlation between the expression levels of WNT5A and WNT10B mRNA and tumor grade in patients with glioma (**A**) Correlation between mRNA expression of WNT5A and tumor grade in patients with glioma as analyzed using CGGA and TCGA data. (**B**) Correlation between mRNA expression of WNT10B and tumor grade in patients with glioma as analyzed using CGGA and TCGA data (*P <0.05, **P <0.01, ***P <0.0001, ****P <0.0001).

Analyzing the mRNA data of 19 WNTs from TCGA and CGGA database, we found that WNT5A mRNA expression level was significantly correlated with the grade of glioma in both TCGA and CGGA databases (Figure 5A); however, WNT10B expression was inversely correlated with the grade of glioma (Figure 5B). Furthermore, the WNTs mRNA expression in glioma of detailed histological classification compared with NB tissues was analyzed with ONCOMINE database. Consistently, higher expression of WNT5A in GBM, Anaplastic Astrocytoma and anaplastic oligodendroglioma was observed; while lower expression of WNT 10B in GBM, oligodendroglioma, astrocytoma and anaplastic astrocytoma was detected. Moreover, WNT7A was also showed lower expressed in astrocytoma than in NB tissues (Table 1).

**Table 1:** Significant differences in the transcription level of WNTs expression between different types of glioma and normal brain tissue using the ONCOMINE database

	Database type	Tumor type	*P*-value	*t*-test	Fold change
WNT5A	Sun brain	Glioblastoma vs. Normal	3.35 ×10^−23^	12.796	2.577
		Anaplastic Astrocytoma vs. Normal	9.86 × 10^−7^	5.963	2.250
	TCGA	Brain Glioblastoma vs. Normal	2.58 × 10^−14^	24.091	4.336
	Lee Brain	Glioblastoma vs. Normal	1.08 × 10^−9^	24.264	14.423
	Bredel Brain 2	Glioblastoma vs. Normal	6.70 × 10^−8^	8.799	3.768
	French Brain	Anaplastic Oligodendroglioma vs. Normal	5.16 × 10^−7^	6.287	3.127
WNT7A	Rickman Brain	Astrocytoma vs. Normal	1.09 × 10^−7^	-6.673	-9.341
WNT10B	Bredel Brain 2	Glioblastoma vs. Normal	1.03 × 10^−12^	-11.601	-6.374
	Sun Brain	Glioblastoma vs. Normal	2.49 × 10^−27^	-15.662	-9.563
		Oligodendroglioma vs. Normal	1.25 × 10^−16^	-10.636	-5.914
		Diffuse Astrocytoma vs. Normal	3.97 × 10^−5^	-6.781	-4.025
		Anaplastic Astrocytoma vs. Normal	7.94 × 10^−8^	-7.364	-6.300
	Rickman Brain	Astrocytoma vs. Normal	3.15 × 10^−5^	-7.069	-2.892
	TCGA Brain	Brain Glioblastoma vs. Normal	1.86 × 10^−9^	-20.916	-7.016

Differences in transcriptional expression were compared by Students’ *t*-test. Cut-off of *P*-value and fold-change were as follows: *P*-value: 0.01, fold-change: 1.5, gene rank: 10%.

### Predicted functions and pathways of WNT5A and WNT10B in patients with glioma

**Figure 6:**
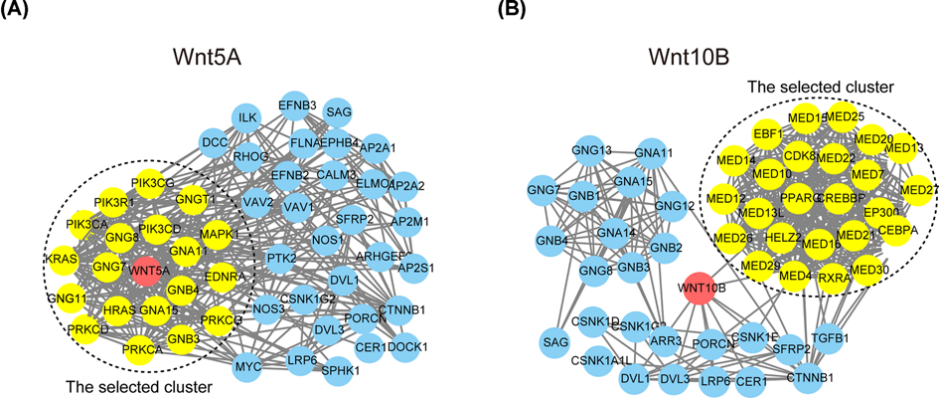
The PPI network complex of WNT5A/10B and the 50 most frequently altered neighbor genes analyzed with String and Cytoscape (**A**) The Module analysis of WNT5A and the 50 most frequently altered neighbor genes. (**B**) The Module analysis of WNT10B and the 50 most frequently altered neighbor genes. The nodes meant proteins; the edges meant the interaction of proteins. Module analysis via Cytoscape software (degree cutoff = 2, node score cutoff = 0.2, k-core = 2, and max. Depth = 100)

As WNT5A and WNT10B were the most correlated with glioma prognosis and clinicopathology, we analyzed the 50 most frequently altered neighbor genes of WNT5A and WNT10B in glioma (Table 2) and constructed integrated networks using String (Figure 6A,B). Then, we applied MCODE and BINGO Apps in Cytoscape for further analysis. Results showed the cluster of WNT5A was correlated with signaling pathway and the cluster of WNT10B was correlated with RNA polymerase II transcription mediator activity (Figure 6A,B).

**Table 2:** Neighboring genes of WNT5A and WNT10B

Gene	Neighbor genes
WNT5A	AP2A1, AP2A2, AP2M1, AP2S1, ARHGEF6, CALM3, CER1, CSNK1G2, CTNNB1, DCC, DOCK1, DVL1, DVL3, EDNRA, EFNB2, EFNB3, ELMO1, EPHB4, FLNA, GNA11, GNA15, GNB3, GNB4, GNG11, GNG7, GNG8, GNGT1, HRAS, ILK, KRAS, LRP6, MAPK1, MYC, NOS1, NOS3, PIK3CA, PIK3CD, PIK3CG, PIK3R1, PORCN, PRKCA, PRKCD, PRKCG, PTK2, RHOG, SAG, SFRP2, SPHK1, VAV1, VAV2
WNT10B	ARR3, CDK8, CEBPA, CER1, CREBBP, CSNK1A1L, CSNK1D, CSNK1E, CSNK1G2, CTNNB1, DVL1, DVL3, EBF1, EP300, GNA11, GNA14, GNA15, GNB1, GNB2, GNB3, GNB4, GNG12, GNG13, GNG7, GNG8, HELZ2, LRP6, MED10, MED12, MED13, MED13L, MED14, MED15, MED16, MED20, MED21, MED22, MED25, MED26, MED27, MED29, MED30, MED4, MED7 PORCN, PPARG, RXRA, SAG, SFRP2, TGFB1

**Figure 7:**
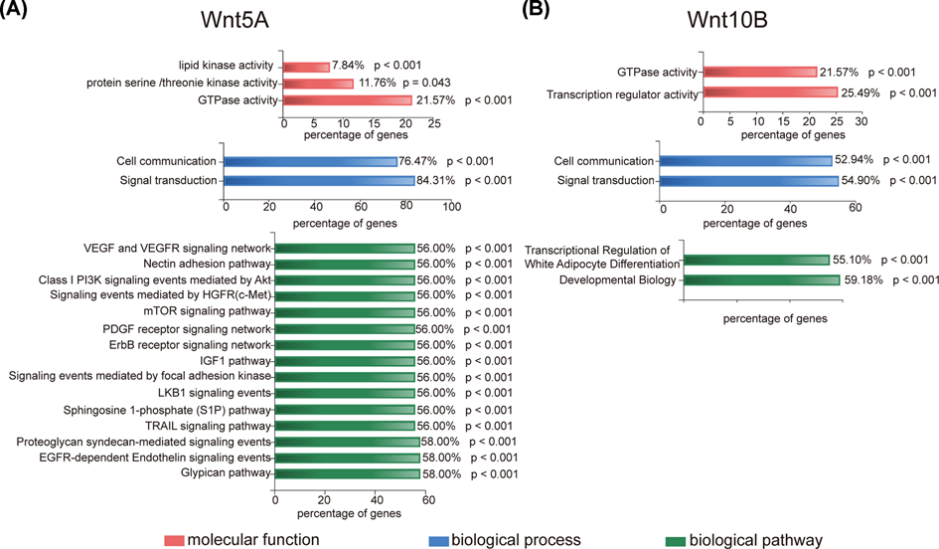
GO enrichment analysis of three main functions of WNT5A and WNT10B, including molecular function, biological process and biological pathway (**A**) GO enrichment analysis predicted three main functions of WNT5A. (**B**) GO enrichment analysis predicted three main functions of WNT10B.

The GO enrichment analysis was then performed to determine the function of WNT5A and WNT10B with neighbor genes in glioma using DAVID. Go analysis predicted that WNT5A regulated molecular functions including GTPase activity, biological processes such as signal translation, and biological pathways such as EGFR-dependent signaling events in glioma (Figure 7A). In addition, GO analysis predicted that WNT10B regulated molecular functions including GTPase activity, biological processes such as signal translation, and biological pathways such as development biology in glioma (Figure 7B).

**Figure 8:**
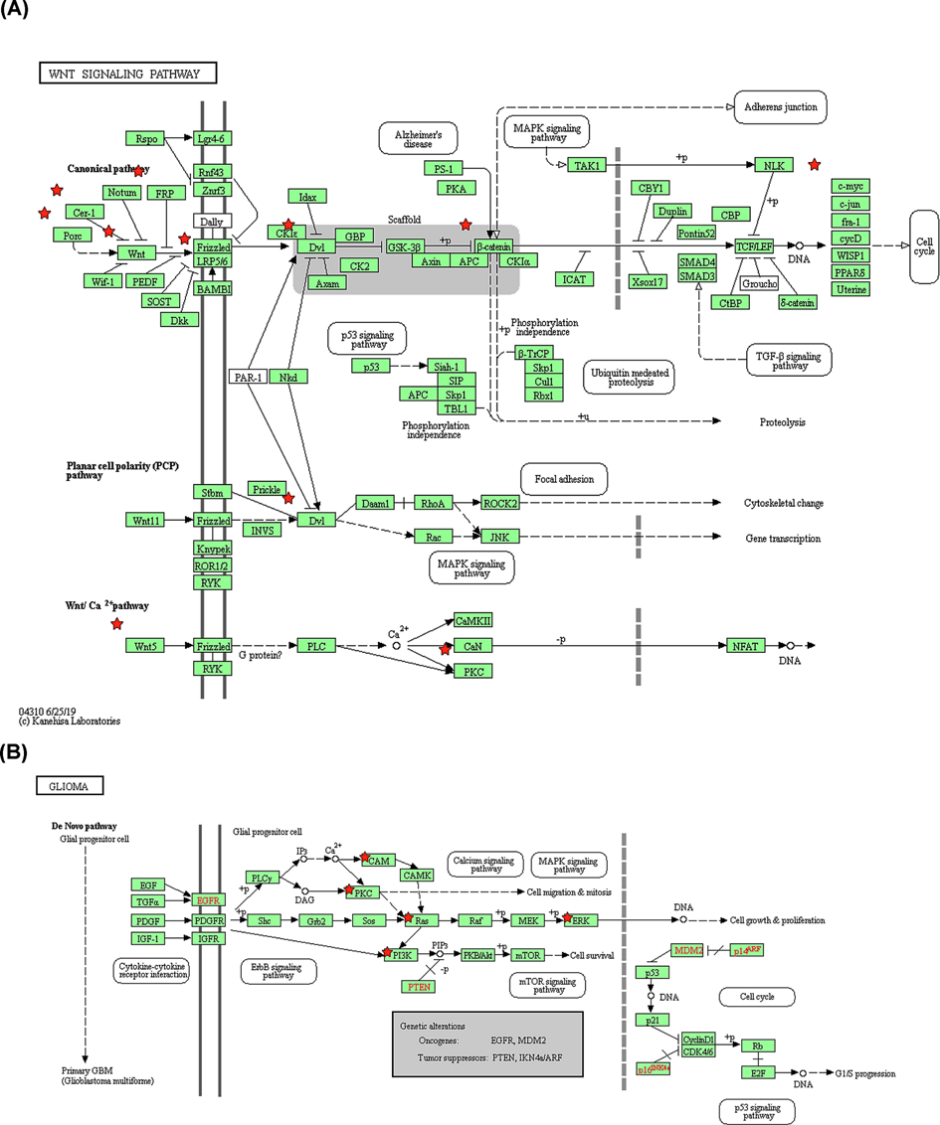
KEGG pathway enrichment analysis of WNT5A/10B and the 50 most frequently altered neighbor genes in glioma analyzed with DAVID (**A**) Three WNT signaling pathways in glioma that WNT5A and WNT10B took part in. (**B**) The associated pathways of WNT5A and the 50 neighbor genes in glioma occurrence and progress.

Finally, KEGG pathway enrichment analysis revealed WNT5A and neighbor genes were essential in the occurrence glioma. WNT5A played role in glioma by taking part in WNT/Ca^2+^pathway and the canonical pathway, however, WNT10B was only ligands of canonical pathway (Figure 8A). In the *de Novo* pathway of glioma occurrence, two crucial oncogenes (EGFR and MDM2) and two important tumor suppressors (PTEN and IKN4a/ARF) were found to be closely correlated with WNT5A in glioma (Figure 8B).

## Discussion

Historically, WNTs were found to regulate many processes in embryonic development, physiology and homeostasis [33,34]. In 1982, for the first time, Nusse and Varmus described WNT-1 (Int-1) as an oncogene in mouse mammary tumors [35]. Up to now, increasing evidences suggested that defective WNT signaling is a causative factor in various cancers including breast cancer [36–38], lung cancer [39], pancreas cancer [40], colorectal cancer [41], hepatocellular carcinoma [42] and as well as glioma [6,8,14]. Despite the fact that some members of WNTs have been confirmed to play crucial roles in glioma, our study is first to investigate expression patterns, prognostic significance, and potential function of all the 19 WNT family members in glioma. We presumed that the findings of this study might contribute to the study of heterogeneity in glioma, and improve the accuracy of prognosis and treatment response and may aid in individualized therapy of patients with glioma. In our study, we found not all WNTs worked equally in glioma. WNT5A was seemed to be a crucial tumor promotor in glioma; WNT10B was seemed to a tumor suppressor in glioma; and other WNTs were not detected statistically different expression and distinct function in glioma.

For WNT5A, significantly higher mRNA expression of WNT5A in glioma was observed compared with NB. The mRNA levels of WNT5A was positively associated with the WHO grade of glioma. The survival analysis revealed that the higher mRNA expression levels of WNT5A was significantly associated with shorter OS in patients with glioma. Among the 19 WNTs, WNT5A is the most commonly studied in glioma, particularly GBM. These results are consistent with earlier studies, WNT5A has indeed been shown overexpression in GBM, and required to maintain the proliferative capability, infiltrative ability, and stem-cell-like characteristics of GBM [8,9,43]. Reis et al. reported elevated expression of WNT5A in glioma comparing with normal tissue [3]. Binda et al. also found the correlation between poor prognosis and WNT5A expression [44]. Hu et al. revealed higher WNT5A expression in recurrent GBM compared with primary GBMs, and pointed out that epigenetic activation of WNT5A triggered stem-cell like GBM invasive growth and differentiation [8]. MicroRNAs (miRNAs) including miR-30a and miR-129-5p has been documented to function as tumor suppressors by targeting WNT5A [45,46].

For WNT10B, in contrast with the results of WNT5B, significant lower mRNA expression of WNT10B in glioma was observed compared with NB. Furthermore, we verified that the mRNA expression of WNT10B was inversely proportional to the WHO grade of glioma. The survival analysis revealed that the higher mRNA expression of WNT10B was associated with better OS of patients. Till date, researches about WNT10B in glioma is few. Only Tayrac et al. reported the under-expression of WNT10B in glioblastoma [47]. According to our study, we suggested a new point that WNT10B might function as a glioma suppressor.

To understand the function and molecular mechanism of WNT5A and WNT10B in glioma, we performed PPI network, GO and KEGG analyses. First, the cluster of WNT5A was correlated with signaling pathway and the cluster of WNT10B was correlated with RNA polymerase II transcription mediator activity in PPI network. Then, GO analysis predicted that WNT5A regulated molecular functions including GTPase activity, biological processes such as signal translation, and biological pathways such as EGFR-dependent signaling events in glioma; differently, WNT10B regulated molecular functions including GTPase activity, biological processes such as signal translation, and biological pathways such as development biology in glioma. Finally, KEGG pathway analysis showed that WNT5A is primarily through the WNT/Ca+ pathway, also activates the canonical pathway [20,48–50] and WNT10B has been only reported in the canonical WNT signaling pathway [51–54]. KEGG pathway analysis also showed that the neighbor genes of WNT5A were widely distributed in multiple pathways in the *de novo* pathways of glioma occurrence, in which crucial oncogenes (EGFR and MDM2) and 2 important tumor suppressors (PTEN and IKN4a/ARF) were closely correlated with WNT5A.

Despite the down-regulation of WNT10B expression in glioma, the most important molecule in the classical pathway, β-catenin is up-regulated in glioma and also reported accumulation in the nucleus of glioma [55,56]. Classical WNT pathway activation may be associated with an increase of WNT5A, but there is no literature reporting WNT5A is the main factor of classic classical pathway activation in glioma. We can just guess WNT5A may cause classic pathways activated in glioma. Furthermore, it is very important to explore WNT5A promoting glioma development through the WNT/Ca+ pathway in glioma more clearly [57]. Our analysis with TCGA and CGGA data showed positive correlation between the expression levels of WNT5A and NFAT subtypes, which are important downstream transcription factors of the non-classical pathway (Supplementary Figure S1). Lastly, the interaction of WNT pathway with many other signaling pathways makes the mechanism of action of WNT pathway more complex [58,59], and our results in Figure 8B provided a direction to explore that.

In the present study, we also revealed that increased mRNA expressions of WNT16 was associated with shorter OS in patients with glioma, while the increased mRNA expressions of WNT3 and WNT5B were associated with longer OS. However, there was not enough evidence for their different expression between glioma and NB tissues.

Although our study provided important insights into the prognostic and research values of WNT5A and WNT10B in glioma patients at the mRNA level, there still are some limitations to the present study. On the one hand, all the data analyzed in our study retrieved from the online databases were RNA expression data, thus, a larger quantity of protein data such as immunohistochemistry is required to validate our findings. On the other hand, though we performed analysis to predict the mechanism and biofunction of WNT5A and WNT10B in glioma, a remarkable amount of work is urgent to investigate the distinct role of WNTs in glioma.

## Conclusion

We systemically investigated the expression profiles and prognostic significance of all the 19 members of the WNT family in glioma. Our results indicated that mRNA expressions of WNT5A and WNT10B were significantly differentially regulated in glioma compared with NB tissues, and associated with pathology and grade of glioma. The study also indicated that WNT5A can serve as a candidate to diagnose and therapy glioma, while WNT10B might be valuable for anti-glioma research. Our data also provided presumed directions to explore interactions in canonical WNT pathway, non-canonical WNT pathway and multiple other signal pathways of interaction in glioma occurrence and development.

## Supplementary Material

Supplementary Figure S1

## Abbreviations

CGGA: Chinese Glioma Genome Atlas
GBM: glioblastoma
GEPIA: Gene Expression Profiling Interactive Analysis
GO: gene ontology
GTEx: Genotype‐Tissue Expression
KEGG: Kyoto encyclopedia of genes and genomes
LGG: low grade glioma
NB: normal brain
OS: over survival
TCGA: cancer genome atlas
WNT: Wingless / Integrated
