# Supplementary material for: Expression profiles and prognostic significance of WNT family members in glioma via bioinformatic analysis
Source: Biosci Rep. 2020 Mar 27;40(3):BSR20194255. doi: 10.1042/BSR20194255 (PMC7103590; doi:10.1042/BSR20194255)
Supplement: Supplementary Figure S1 [file BSR-2019-4255_supp.pdf]

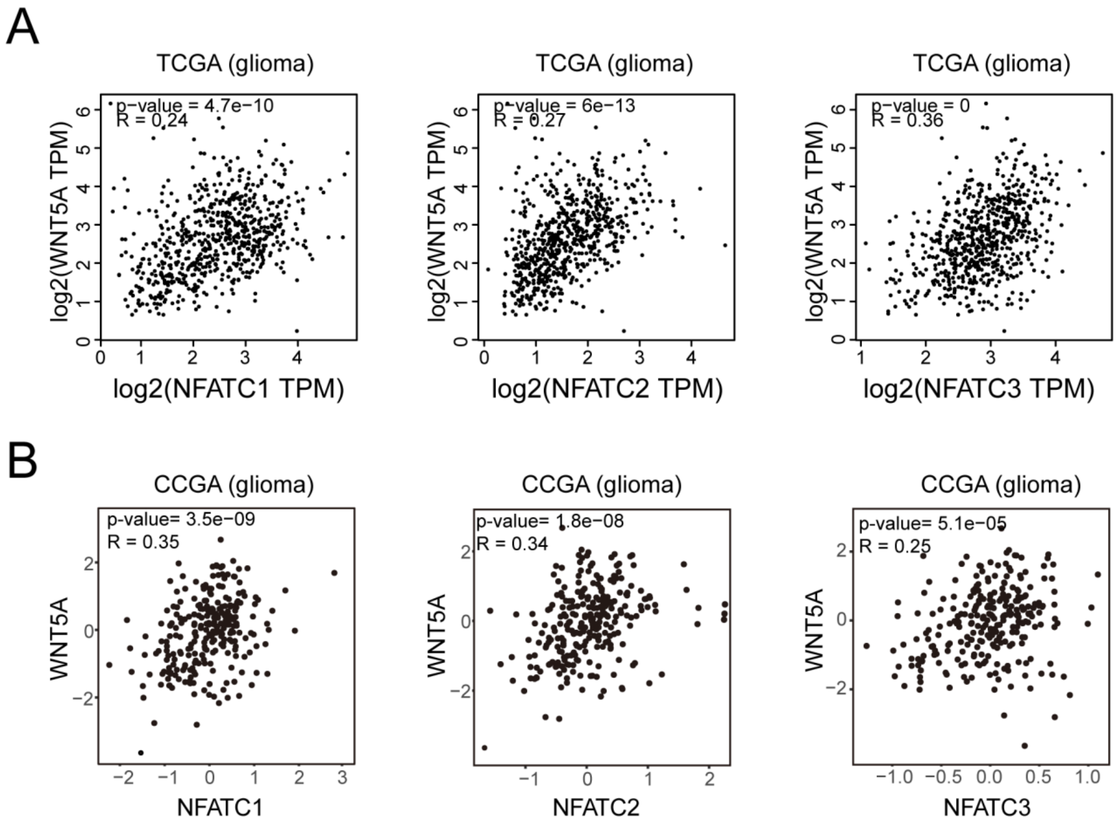
A
TCGA (glioma)
p−value = 4.7e−10
R = 0.24
log2(WNT5A TPM)
log2(NFATC1 TPM)
TCGA (glioma)
p−value = 6e−13
R = 0.27
log2(WNT5A TPM)
log2(NFATC2 TPM)
TCGA (glioma)
p−value = 0
R = 0.36
log2(WNT5A TPM)
log2(NFATC3 TPM)
B
CCGA (glioma)
p-value= 3.5e−09
R = 0.35
WNT5A
NFATC1
CCGA (glioma)
p-value= 1.8e−08
R = 0.34
WNT5A
NFATC2
CCGA (glioma)
p-value= 5.1e−05
R = 0.25
WNT5A
NFATC3

Supplementary figure 1: (A) Positive correlation between the expression levels of WNT5A and NFAT subtypes analyzed with TCGA data. (B) Positive correlation between the expression levels of WNT5A and NFAT subtypes analyzed with CCGA data.
